# Editorial: The role of interplay between metabolism and chromosomes in tumorigenesis

**DOI:** 10.3389/fcell.2022.981075

**Published:** 2022-08-10

**Authors:** Lutao Du, Yuanyuan Lu, Jiayi Wang

**Affiliations:** ^1^ Department of Clinical Laboratory, The Second Hospital of Shandong University, Jinan, Shandong, China; ^2^ Shandong Provincial Clinical Medicine Research Center for Clinical Laboratory, Jinan, Shandong, China; ^3^ State Key Laboratory of Cancer Biology, National Clinical Research Center for Digestive Diseases, Xijing Hospital of Digestive Diseases, Fourth Military Medical University, Xi’an, China; ^4^ Department of Laboratory Medicine, Shanghai Chest Hospital, Shanghai Jiao Tong University, Shanghai, China; ^5^ Shanghai Institute of Thoracic Oncology, Shanghai Chest Hospital, Shanghai Jiao Tong University, Shanghai, China

**Keywords:** non-coding RNA, post-translational modification, epigenetic regulation, glycolysis, m6A modification, cancer, ceRNA

## Introduction

Tumor is one of the biggest threats to human health. Genetic and epigenetic modifications in chromosome contribute to altered gene expression in tumor formation and development (Lee and Kim, 2022). In addition, altered metabolism such as activated glycolysis pathway is an essential feature of tumor to provide energy and structural resources for tumor cell proliferation (Altman et al., 2016). Previous studies have preliminarily confirmed the relationship between chromosome and metabolism, and the role of them in jointly promoting the development of tumor. For example, metabolites produced in tumor cells can affect enzyme activity of epigenetic modification to regulate gene expression (Miranda-Goncalves et al., 2018); some metabolites directly modify chromatin as substrates, resulting in chromatin structural abnormalities and modifications (Dai et al., 2020). In addition, genetic mutations associated with chromatin instability can lead to changes in enzyme activity resulting in the production of tumor-promoting metabolites (Kim and Yeom, 2018). Accumulation of harmful metabolites can trigger further mutations in chromosomes (Janke et al., 2015; Pavlova and Thompson, 2016; Pavlova et al., 2022). In our Research Topic, we further refined the relationship between chromosomes and metabolism from the following aspects, and clarified their cancer-promoting roles.

## Metabolic regulation of non-coding RNA

Non-coding RNA originates from chromosomes, and exerts cis-regulatory effects at the chromosomal level (Quinn and Chang, 2016). N^6^-methyladenosine (m^6^A) is the most abundant internal modification of RNA in eukaryotic cells, and has gained increasing attention in recent years. m^6^A can affect RNA function by affecting RNA processing, nuclear export and RNA translation to decay (Huang et al., 2020). In addition, m^6^A affects RNA stability and thus affect cell function. For example, in dendritic cell, CCR7 chemokine receptor upregulates long non-coding RNA Dpf3 via removing m^6^A modification to prevent RNA degradation. Dpf3 directly binds to and suppressed HIF-1α-dependent glycolysis (Liu et al., 2019). In our Research Topic, Liu et al. and Liang et al. reported that m^6^A modifications of long non-coding RNA DUXAP8 and LINC00106 both upregulate their stability, and then increase the migration, invasion, and metastasis characteristics of tumor cells and make tumor cells acquire chemotherapy-resistant properties. In addition, Liu et al. found that a glycolysis promoting gene SLC2A1 is closely associated with multiple m^6^A-related genes. These data suggested the close relationship between metabolism and pan-m^6^A modification system.

Competing endogenous RNAs (ceRNAs) are transcripts that can regulate each other at post-transcription level by competing for shared miRNAs (Qi et al., 2015). ceRNA network is an important way for non-coding RNAs to exert their functions (Tay et al., 2014). For example, HMGB1 mRNA has been found to promote the expression of RICTOR mRNA as the ceRNA through competitively binding with the miR-200 family, especially miR-429, then promotes glutamine metabolism and impedes immunotherapy by PD-L1+ exosomes activity (Wei et al., 2021). In our Research Topic, we also provide evidences that ceRNAs regulate tumor-related metabolism. The above mentioned long non-coding RNA DUXAP8, LINC00106 can both absorb microRNA to enhance the malignant phenotype of HCC cells. In addition, Lai et al. reported that LINC01572 and Wang et al. reported that LUCAT1 both act as ceRNAs to promote glycolysis in tumor cells. The above-mentioned glycolysis promoting gene SLC2A1 is also bio-informatic analyzed to be associated with ceRNA regulatory network. Li et al. reported that MTFR1 is a promoter of tumor progression and glycolysis by activating the AMPK/mTOR signalling pathway in LUAD. The MTFR1 negative regulator is miR-29c-3p, but the upstream ceRNA is not reported. We also published a review written by Xu et al. reported the role of non-coding RNA in cerebrospinal fluid. This review elucidates the brain tumorigenic mechanisms of non-coding RNAs in cerebrospinal fluid (mainly through m^6^A modification and ceRNA regulation), and concludes the research results of various non-coding RNAs as diagnostic, prognostic markers and therapeutic agents.

Altogether, the above articles describe that the m6A modification of non-coding RNAs to upregulate its stability, or through the downstream ceRNA network to activate glycolytic metabolism to play a tumor-promoting role.

## Metabolic regulation of anticancer agent

Epigenetic regulated proteins such as histone modification readers and writers have many inhibitors/stimulators that regulate tumor metabolism by affecting epigenetics (Wimalasena et al., 2020). In our Research Topic, Qi et al. reported that Paris Saponin II inhibits human head and neck squamous cell carcinoma cell proliferation and metastasis by inhibiting the expression NOS3 and the nitric oxide metabolic pathway.

## Metabolic regulation by post-translational modification

There are numerous examples of post-translational modifications regulating metabolism, such as protein phosphorylation, O-Glycosylation, methylation and crotonylation. A recent study reported that UCP1 is an important thermogenic protein in brown adipose tissue, and there are two key succinylation sites on UCP1 that are critical for UCP1 stability and activity. Loss of UCP1 succinylation results in impaired mitochondria respiration, defective mitophagy, and metabolic inflexibility (Wang et al., 2019). In our Research Topic, Wang et al. reported that ataxia-telangiectasia mutated deficiency firstly augments the phosphorylation of Mad1 Ser214, and promotes its heterodimerization with Mad2. Subsequently, Mad2 is phosphorylated at Ser195. This Mad2 phosphorylation decreases DNA damage repair capacity and is related to the radiotherapy resistance.

## Epigenetic regulation by glycolysis

Glycolysis also has important effects on epigenetics. For example, a large amount of lactic acid is produced through glycolysis, and then protein lactylation occurs (Zhang et al., 2019). H3K18 lactylation elevates the expression of METTL3, which in turn leads to the activation of JAK/STAT signaling pathway, and further promotes cancer (Xiong et al., 2022). In our Research Topic, Shen et al. reported that in the hypoxic HCC microenvironment induced by glycolysis, ubiquitin E3 ligase ring finger protein RNF146 is transcriptionally activated by HIF-1α and HIF-2α. RNF146 promotes PTEN ubiquitination and degradation and stimulates AKT/mTOR pathway, thereby promotes HCC cell proliferation.

## Epigenetic modification-related metabolic markers

Markers simultaneously associated with epigenetics and metabolism are helpful for the treatment and evaluating prognosis of tumors. Existing markers include *TET2*, *IDH1/2*, *DNMT3A* recurrent mutations and so on (Nebbioso et al., 2018). Via metabolomic and transcriptomic analysis, Shan et al. found that 5-hydroxylysine and 1-methylnicotinamide are most likely to be keloid severity indicators.

In our Research Topic, a review written by Huo et al. details the relationship between epigenetic modification and metabolism in tumors. Firstly, epigenetic modifications such as deoxyribonucleic acid and ribonucleic acid methylation, non-coding ribonucleic acids and histone modifications regulate the metabolic remodeling of tumors, including glucose and lipid metabolism. In the contrary, intermediates produced by metabolism often participate in epigenetic regulation by serving as substrates or cofactors for epigenome-modifying enzymes. Therefore, the authors suggested that the metabolism-epigenome axis must be considered while approaching cancer biomarker studies.

Collectively, our Research Topic elaborated the influence of chromosomal substances such as non-coding RNA and its m^6^A modification, as well as post-translational modification of proteins on tumor glycolysis and other metabolism, and analyzed the influence of glycolysis on ubiquitination modification. Some epigenetic modified-related metabolic markers were found. Therefore, the close relationship between chromosome and metabolism is very important for the occurrence and development of tumors. In future research and clinical treatment, we need to explore the relationship between chromosome and metabolism in more detail.

**FIGURE 1:**
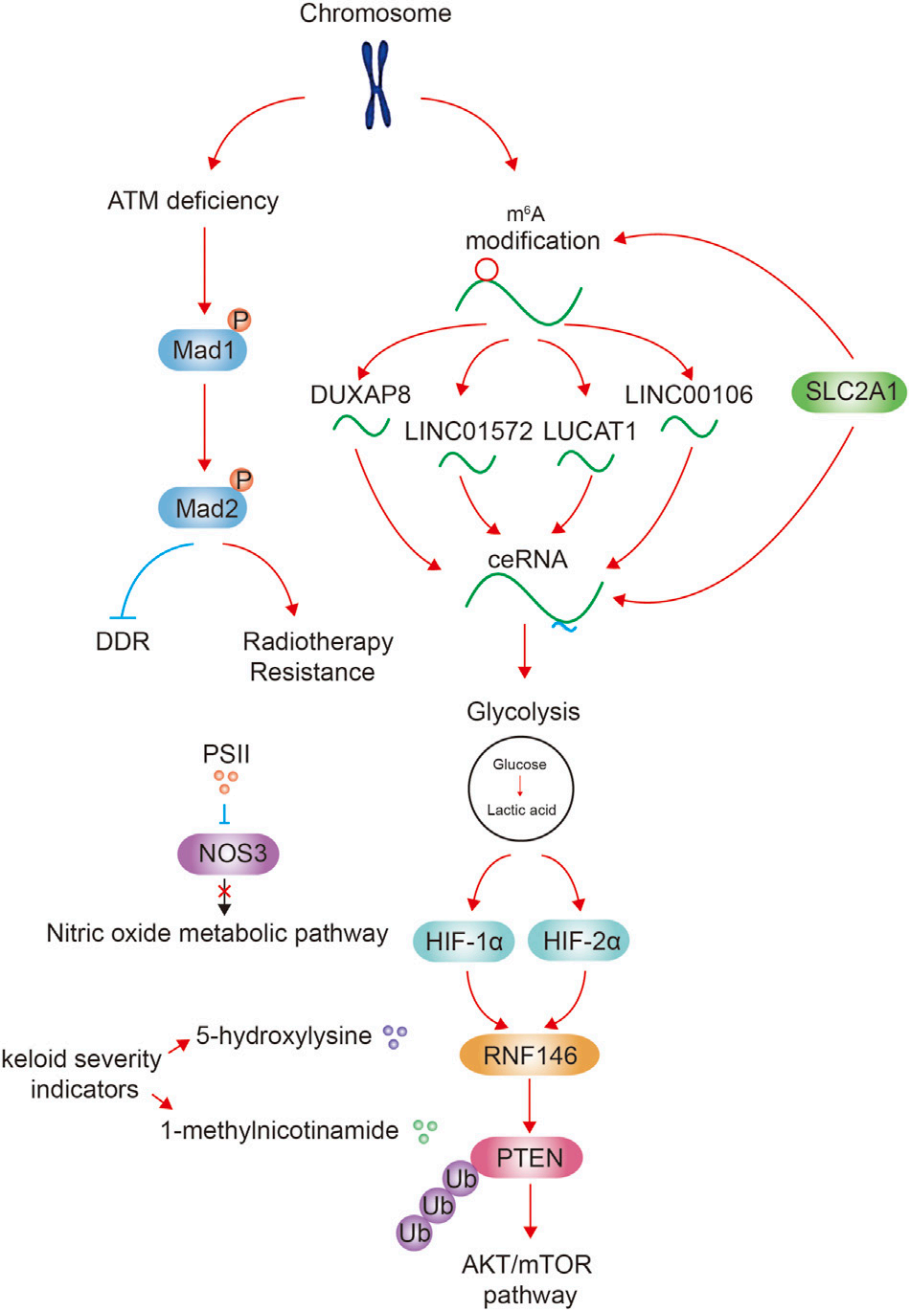
XXX.
